# Self-regulated learning microanalysis for the study of the performance of clinical examinations by physiotherapy students

**DOI:** 10.1186/s12909-020-02149-7

**Published:** 2020-07-22

**Authors:** Raquel I. Medina-Ramírez, D. David Álamo-Arce, Felipe Rodriguez-Castro, Dario Cecilio-Fernandes, John Sandars, Manuel J. Costa

**Affiliations:** 1 Health Sciences School, University of Las Palmas, Las Palmas, Spain; 2 Health Science, Blas Cabrera Felipe Street, without number 35016 – Las Palmas of Gran Canaria, Canary Island, Spain; 3 Department of Medical Psychology and Psychiatry, School of Medical Sciences, University of Campinas, Campinas, São Paulo, Brazil; 4 Health Research Institute, Faculty of Health, Social Care and Medicine, Edge Hill University, St Helens Road, Ormskirk, UK; 5 Life and Health Sciences Research Institute (ICVS), School of Medicine, University of Minho, Braga, Portugal

**Keywords:** Self-regulated learning, Physical therapy techniques, Clinical skills, Assessment process, Health student

## Abstract

**Background:**

Students require feedback on their self-regulated learning (SRL) processes to improve the performance of clinical examinations. The key SRL processes used by students can be identified by SRL-micro-analysis but, this method has not been previously applied to physiotherapy students. The aim of this pilot study was to test a research design that might allow the evaluation of the potential usefulness of SRL microanalysis for the identification of key SRL processes used by physiotherapy students during the performance of a clinical examination skill. The objectives of the pilot study were: 1) to evaluate whether SRL-microanalysis could identify differences in the use of SRL processes between successful and unsuccessful students; 2) to evaluate the reliability of SRL microanalysis ratings produced by different assessors.

**Methods:**

SRL-microanalysis was used with second year physiotherapy students of a Spanish university (*n* = 26) as they performed a goniometric task. The task required students to obtain a goniometric measurement of the shoulder joint of a peer. Two assessors evaluated student performance and conducted the SRL- microanalysis with all students. An analysis of inter-rater reliability was performed to evaluate the degree of agreement between assessors.

**Results:**

The SRL-microanalysis revealed differences in the use of key SRL processes between successful (*n* = 15: 57.0%) and unsuccessful performers (*n* = 11: 43.0%): The differences were particularly evident in strategic planning and self-monitoring skills. There was good inter-rater reliability for scoring of strategic planning (k = 0.792), self-monitoring (k = 0.946) and self-evaluation (k = 0.846).

**Conclusion:**

The use of SRL microanalysis characterized the key SRL processes of physiotherapy students performing a clinical skill with reliability between the assessors. This pilot study supports the potential usefulness of SRL-microanalysis for the identification of key SRL processes in physiotherapy education. Therefore, this study paves the way to the development of a full study, with a larger number of students and more diverse clinical tasks, to evaluate the SRL processes in successful and unsuccessful students.

## Background

There is strong evidence across diverse contexts, from academic studies to music education and athletic training, that self–regulated learning (SRL) has an important contribution to both understanding and to inform feedback for enhancing performance [1–4].

SRL is a meta-cognitive process that has been defined as ‘self-generated thoughts, feelings, and actions that are planned and cyclically adapted to the attainment of personal goals [2]. Learners who self-regulate engage in goal-directed behaviours, use specific strategies to attain goals, and modify their goal-directed behaviours or strategies to optimise learning [2]. One of the most widely applied models of SRL was proposed by Zimmerman and is grounded in social cognitive theory [2, 5]. This model consists of 3 cyclical and iterative phases: forethought, performance, and self-reflection [6]. In the forethought phase, which takes place before the start of the task, learners anticipate the nature and complexity of the task at hand, set goals, and make specific plans to ensure appropriate performance [5]. The impetus for a learner to invest the necessary effort to engage in self-regulation is determined by self-motivation beliefs, such as self-efficacy, goal orientation, and task interest or value [1]. In the performance phase, self-regulated learners focus on monitoring and adjusting their actions. The strategies used include attention focusing, relaxation, positive self-talk, and mental rehearsal of the steps of the task [7]. In the self-reflection phase, after the task is concluded, learners self-evaluate their use of SRL processes to achieve the task and reflect on whether these processes need to be modified for enhancing future performance (7).

The use of SRL processes by learners are not amenable to direct observation but there are assessments which capture the key SRL processes that individuals employ to perform a specific task [8]. Such assessments provide useful information to enhance feedback to the learner [9]. SRL-microanalysis is designed specifically to evaluate how learners self-regulate across the three phases of the SRL cycle [10] using “think aloud protocols” during real-time observation [1, 8]. At predetermined moments, answers to questions related to the forethought, performance, and self-evaluation phases are collected and subsequently analysed [11]. SRL-microanalysis contrasts with approaches that rely solely on questionnaires, which are not designed to capture the entire SRL cycle and are subject to bias related to the beliefs of an individual in self-efficacy or attribution bias [12].

Cleary and Sandars have investigated the use of key SRL processes in medical students performing the clinical skill of venepuncture. They found that students with higher levels of strategic thinking before, during, and after the venepuncture, performed better than those with low levels of strategic thinking [13]. A narrative review of published meta-analyses of feedback interventions in education and a systematic review of effective remediation interventions in medical education have highlighted the importance of enhancing performance feedback with feedback about the use of key SRL processes by students [14, 15].

Despite the well-established importance of SRL in diverse educational contexts, including medical education, it is unknown whether poor performance of clinical skills in physiotherapy students may also be associated with difficulties in SRL. Therefore, before conducting a full study to address this gap, we developed a pilot study to evaluate the potential usefulness of SRL microanalysis in physiotherapy students. Pilot studies provide essential information about whether the rationale for a study and the proposed methods are inappropriate or overly complicated [16]. Our pilot had a focus on (a) whether our SRL-microanalysis method, can identify differences in the use of planning, monitoring and self-evaluation, between successful and unsuccessful students performing a clinical task and (b) the reliability of the SRL-microanalysis scoring made by different assessors of the students’ use of key SRL processes as they performed a clinical task.

The aim of this pilot study was to test a research design that may clarify the potential usefulness of SRL microanalysis for the identification of key SRL processes used by physiotherapy students during the performance of a clinical examination skill. The objectives of the pilot study were: 1) to evaluate whether SRL-microanalysis could identify differences in the use of SRL processes between successful and unsuccessful students; 2) to evaluate the reliability of SRL microanalysis ratings produced by different assessors.

## Methods

### Participants and setting

Participants were undergraduate second year physiotherapy students at the Faculty of Health Sciences, University of Las Palmas, in Gran Canaria, Spain. Students were recruited at the conclusion of a lecture by the first author (RM). The general nature of the study was explained without passing on specific information about SRL. All participants had successfully completed the “*Valoración en Fisioterapia I*” (Assessment in Physiotherapy 1)*-*UNESCO code 3211.11, within the previous 3 months, in which they had performed joint goniometric measurements similar to the clinical skill task used in this study.

### The goniometric task

We chose goniometry for our study as it is a common clinical skill task. It is also well-defined within international physiotherapy curricula, for example, in the Canadian physiotherapy curriculum [17]. It consists of assessing the range of a joint’s motion by measuring the angle of motion [18]. In this study, students were instructed to obtain a goniometric measurement of the shoulder joint of a peer. This task included several actions: positioning of the peer into a correct posture, setting the goniometer in the correct position, moving the joint correctly through its range of motion, and obtaining the measurement of the range of the angle of shoulder flexion [18].

### The SRL-microanalysis protocol

The SRL-microanalysis protocol followed guidelines used in medical education [7]. Before the start of the interview, the interviewer described the task to the participant. The participant would then judge their ability to perform the task on a scale from 0 to 10, and answer the strategic planning question: “Do you have any particular plans about how you will obtain the measurement?*”.* After answering the question, the participant would perform the task. After positioning the goniometer and prior to making any joint movement in the peer, the participant answered the self-monitoring question: “Do you think you have performed a flawless process so far or have you made any mistakes? Tell me about them”. Finally, upon task completion, two self-evaluation questions to identify self-calibration were posed. Accurate self-calibration of performance is essential to initiate any change in future performance [19]. The first question was “How satisfied are you with your current performance?” on a scale from 0 to 10 [20]. The second was an open question: “What criteria did you use to determine your satisfaction?”. Finally, students were asked to judge their performance on a scale from 0 to 10 [20] (See Table 1).

**Table 1:** SRL Microanalytic Assessment protocol

SRL Phase	SRL Sub process	Measure/Questions	Timing of administration	Coding Scheme
Forethought	Self-efficacy Pre-Task	Scale 0–10	Pre-task	0–10
	Strategic Planning	Do you have any particular plans for how to take data about the joint grades?	Immediately preceding the first attempt to take the measure.	1) Patient focus
				2) Technique focus
				3) Patient care and technique focus
				4) No plan
				5) Do not know
Performance	Self-monitoring	Do you think you have performed a flawless process thus far or have you made any mistake? Tell me about them.	After the measure began but prior to obtaining goniometric grades.	1) Not aware of any mistake
				2) Procedural mistake
				3) Non-procedural mistake
				4) Do not know
Self Evaluation	Satisfaction	How satisfied are your current performance?	After the task was completed.	0–10
		Scale 0–10		
	Self-evaluation	What criteria did you use to determine your satisfaction?	After satisfaction question	1) Lectures
				2) Practical lessons
				3) Lectures and practical lessons
				4) Other factors
				5) Do not know
	Self-efficacy Post-Task	Scale 0–10	After self- evaluation question.	0–10

### Data collection

This study received ethical approval from The Ethical Committee of Human Research of the ULPGC, reference CEIH-2018-01**.** All participants gave written informed consent before data collection began.

Prior to the observations two experienced physiotherapists (RM and DA) agreed on the expected standard of performance for the task. Independently, they marked the performance of each student as successful or unsuccessful. All answers were audio-recorded and transcribed by the first author (RM). Each SRL microanalysis session lasted from 3 to 6 min.

### Data analysis

The coding scheme was established in advance following the guidelines for SRL microanalysis (for more information please see [7]). Verbal responses were recorded and subsequently coded into categories using a framework from prior research [7, 10, 11]. The responses to the open questions were coded independently [13] by two authors (RM and DA). The inter-rater agreement was calculated using kappa coefficients. Differences in coding between examiners across all SRL measures were resolved through discussion among the authors (RM, DA and MJC).

Answers to open question were coded into the following categories:

- Strategic Planning: 1) Positioning the patient (patient focus); 2) Technical performance using the goniometer (technique focus); 3) Patient and technique combined; 4) Without a plan; 5) Do not know
- Self-monitoring: 1) not aware of any mistakes; 2) mentions procedure related mistakes; 3) non-procedure related mistakes; 4) do not know.
- Self-Evaluation: 1) learning originating from theoretical lectures; 2) learning originating from practical sessions; 3) learning originating from both theoretical and practical sessions; 4) Other; 5) Do not know.

To investigate the pre and post difference between students’ self-evaluation of performance (calibration), we calculated t paired sample. For the quantitative analysis, we used SPSS 21.0.

## Results

### Recruitment

The study enrolled 26 students, 19 were female (73.7%) and 7 were male students (26.9%). They represented 38.8% of the second-year physiotherapy class.

### Task performance

There were 15 successful students (57%) and 11 (43%) unsuccessful students on the goniometric task. There were proportionally fewer female students in the unsuccessful group (*n* = 7) 63.6% compared to the successful group (*n* = 12) 80%.

### SRL processes

#### (a) Forethought phase

In the forethought phase, most successful students [14:15 (93%)] had planned the task ahead and only one student stated no planning for performance. The plans described by the successful students fell into three categories: positioning the patient (patient focus) and correct technical performance using the goniometer (technique focus) combined (*n* = 6, 40%), technique focus (*n* = 3, 20%), or patient focus alone (*n* = 5, 33.3%).

We present three illustrative statements on focusing on the technique made by successful students:

**017***: “I think I have a plan ... I put the goniometer first. I would ask him to raise his arm and measure it. “.*

**020***: “First I place the stretcher at a comfortable height, I ask the patient to get into the most comfortable position and explain what he has to do. He should be comfortable”.*

**015***: “Yes, I have a plan. First, I place the patient in a supine position, to be comfortable and I adjust the stretcher. Then, I put the axis of the goniometer on the lateral side of the humerus, the fixed arm parallel to the midline of the humerus... The fixed one remains there, and another moves parallel to the midline of the humerus. And I ask him for the flexion movement. And I measure it”.*

In the forethought phase, six (54.5%) unsuccessful students were unable to explain their action plan or stated they had no strategy for performing the task. These students were categorised as “Without a plan”. The plans of unsuccessful students could also be categorized into technique (*n* = 2, 18.9%), patient (*n* = 1, 9.1%) or technique and patient (*n* = 2, 18.9%).

#### (b) Performance phase

The narratives of successful students were very detailed, revealing attention to the details of their performances. Successful students mentioned they were under the impression they had committed a mistake (*n* = 9, 60%), related to the procedure (e.g., incorrect/imperfect positioning of the goniometer (*n* = 6, 40%) or to their own posture or the position of the bed (non-procedural) (*n* = 3, 20%). There was a single successful student who did not acknowledge to have self-monitored their performance. In contrast, none of the unsuccessful students could recognize their mistakes. Answers were divided in two categories: those who explicitly mentioned they had made no mistakes (*n* = 5, 46%) or those who were unable to answer the question (*n =* 6, 54.5%). This finding suggests that the student had internalised the task to a level of expertise and the key SRL processes had become routinized. For more SRL microanalysis procedure details see Tables 2 and 3.

**Table 2:** Qualitative variables: Strategic planning, Self-monitoring and Self-evaluation

	QUALITATIVE ANALYSIS	SUCCESSFUL (n)	UNSUCCESSFUL (n)	TOTAL
STRATEGIC PLANNING COODING	Patient care	5	1	6
	Technique	3	2	5
	Patient care and technique	6	2	8
	No plan	1	6	7
	Do not know	0	0	0
	TOTAL	15	11	26
MONITORING CODING	Not aware of any mistake	5	5	10
	Procedural mistake	6	0	6
	Non-procedural mistake	3	0	3
	Do not know	1	6	7
	TOTAL	15	11	26
SELF-EVALUATION CODING	Lectures	7	2	9
	Practical lessons	2	0	2
	Lectures and practical lessons	1	3	4
	Other	2	3	5
	Do not know	3	3	6
	TOTAL	15	11	26

**Table 3:** Examples quotes in each phase differentiated by successful and unsuccessful students

PHASE	CODING SCHEME	EXAMPLES SUCCESSFUL QUOTES	EXAMPLES UNSUCCESSFUL QUOTES
**FORETHOUGHT PHASE: Do you have any particular plans for how to take data about the joint grades?**	1) Patient interaction/care	*020. First I place the stretcher at a comfortable height, I ask the patient to get into the most comfortable position and explain what he has to do. He should be comfortable”.*	*013. “I have to tell the patient what I am going to do, put him in a good position and perform the task.”*
	2) Technique	*017.”I think I have a plan ... I put the goniometer first. I would ask him to raise his arm and measure it. “*	*011. “Yes, I follow the bony regions and how is the movement to apply the tool”.*
	3) Patient care/ technique	*015.”Yes, I have a plan. First, I place the patient in a supine position, to be comfortable and I adjust the stretcher. Then, I put the axis of the goniometer on the lateral side of the humerus, the fixed arm parallel to the midline of the humerus.. And I measure it”*	*003. “First, I prepared the patient, and then I allocate correctly the goniometer”*
	4) Any plan	*030. “I have no plan right now”*	*021.”I am not thinking about a plan right now”*
	5) Do not know	*No examples*	*No examples*
**PERFORMANCE PHASE: Do you think you have performed a flawless process thus far or have you made any mistake? Tell me about them.**	1) Not aware of any mistake	*006: “I made mistakes, I think ... I have to put the goniometer in this way… I am not considering the alignment of the goniometer...”*	*009. “No, it is correct”*
	2) Procedural mistake	*026: “I think I am making mistakes in my posture ... maybe my leg on the stretcher.”*	*No examples*
	3) Non-procedural mistake	*030. “I thin it is correct”*	*No examples*
	4) Do not know	*012. “I am not sure…I do not know”*	***07:****“I do not know if I have made any mistakes...”*
**SELF-EVALUATION PHASE: What criteria did you use to determine your satisfaction?**	1) Lectures	*026: “what I remember from lectures…I should put it in the right way and if it should go in the arm or move or not...”*	*009. “The knowledge learned in lectures”*
	2) Practical lessons	*030. “The concept learned in the practical lessons and practical exams”*	*No examples*
	3) Lectures/ practical lessons	*020. “In what I have learned in lectures and practical lessons during the year”*	*013. “Beacuse I have learnt how to do it in lectures and practical lessons”*
	4) Other factors	*016. “First of all, I were insecure with the goniometer and then I realised my mistakes..”*	*007. “I observed my performance and I realised my mistakes”*
	5) Do not know	*015. “I do not know exactly..”*	*021. “I do not know….I do not remember…”*

We present two illustrative statements of self-monitoring and awareness of procedural mistakes by successful students;

***06****: “I made mistakes; I think ... I have to put the goniometer in this way… I am not considering the alignment of the goniometer...”*

***26:****“I think I am making mistakes in my posture ... maybe my leg on the stretcher.”*

#### (c) Self- evaluation phase

There was little difference in answers by successful or unsuccessful students to the question on self-evaluation. Successful students (*n* = 7, 47%) were mostly focused on the importance of paying attention in lectures. An illustrative statement from a successful student:

**026***: “what I remember from lectures…I should put it in the right way and if it should go in the arm or move or not...”*

The median scores of successful and unsuccessful students’ self-evaluation judgments of performance (calibration) were, respectively, 6 and 8. After the task, the judgment scores were higher for successful students (median = 8) than unsuccessful students (mean = 7). The differences between the judgment of performance scores pre and post task were statistically significant (t = 2.613, *p* = .015) with a medium effect size (*r* = 0.45) [21].

There were three unsuccessful students with high judgment of performance scores before starting the task who were unable to complete the task. After the task, two of these students reduced their judgment. The other student maintained the same judgment after an unsuccessful performance. Although the student who maintained a high judgment of performance had a planned the performance, the student lacked awareness of mistakes when self-monitoring his performance. These findings suggest that the student was overconfident and poorly calibrated in his initial and final judgments in relation to his performance on the task.

The satisfaction scores were higher in successful students (mean = 8.07), than in unsuccessful students (mean = 6.27). This difference between successful and unsuccessful students was significant (t = 2.663, *p* = 0.014).

### Inter-rater reliability

The inter-rater kappa coefficients for strategic planning (0.792), self-monitoring (0.946) and self-evaluation (0.846) were high. For internal consistency, an alpha-Cronbach coefficient of 0.846 was obtained for self-judgment prior and post task, and satisfaction post-task.

## Discussion

This pilot study suggests that a full study with the same research design applying SRL microanalysis to evaluate the use of SRL by physiotherapy students as they perform a clinical skill, may uncover SRL difficulties of physiotherapy students that would otherwise be unnoticed. As expected, we found differences between unsuccessful and successful students in strategic approaches to goniometric measurements, namely in strategic planning and self-monitoring.

The differences between successful and unsuccessful students in their use of strategic planning and self-monitoring processes are in line with previous findings in medical students [10, 11, 13]. For example, in a venepuncture simulation context, Sandars and Cleary found differences in strategic approaches of Year 2 medical students [13]. The two main differences between successful and unsuccessful students were similar to our findings, with an overall difference in strategic planning and self-monitoring. The wider literature also shows that individuals who focus on their planning make better adjustments during the task, compared to those who do not plan the activity [22]. This study also relates to research in other domains like sports performance, in professional development, in musician’s performance and in medical education [3, 11, 23, 24].

Interestingly, before the performance of the task, judgment of performance was higher in unsuccessful students than successful. The literature suggests that this lack of calibration between perceived success in performing a task and their actual performance is greater in unsuccessful students than successful students [25]. The Dunning-Kruger effect, in which unsuccessful students judge their knowledge or performance as better than successful students, also applies [26]. One explanation may be that unsuccessful students think that they have all the necessary knowledge and skills, leading to premature closing of studying and practicing.

To our best knowledge, researchers have not yet applied SRL microanalysis techniques to understand students’ use of SRL processes during the performance of clinical skills in the physiotherapy context. Although we found interesting differences between the use of SRL processes between unsuccessful and successful students, our primary focus has been on methodological development reflecting the breadth of use of key SRL processes during a clinical task. First, the data suggest that SRL-microanalysis may be carried out independently by multiple assessors with high inter-rater reliability. Second, the recruitment of students was successful with around 40% of students’ participation, suggesting that scaling the number of participants should be possible. The protocol was easy and fast, taking around 8 min. The questions were short and the answers were succinct, which in turn facilitated the transcription and analysis of the data.

The incorporation of SRL-microanalysis in the diagnosis of difficulties in student performance of clinical skills could potentially enhance the effectiveness of remedial programs, by informing and directing the feedback to aspects that students need to address to enhance their performance [9, 14] . The assumption that students can develop key SRL processes is aligned with the idea that SRL interventions are one form of helping students develop as independent, lifelong learners [23].

### Weakness and future research

This study shares the weaknesses of any pilot study in terms of the generalizability of findings. Our study was restricted to a small sample from one institution and, in terms of clinical skills it was restricted to goniometry of one joint. However, the consistency of our findings with previous research suggests that similar findings may also occur with studies performed on other clinical skills in physiotherapy.

This pilot study was an attempt to understand whether the use of SRL-microanalysis would add value to the identification the key SRL processes, particularly when students were unsuccessful. Our findings support the potential of applying SRL microanalysis for the characterization of the use of key SRL processes by physiotherapy students while performing a clinical skill. Important aspects of the potential usefulness of the SRL microanalysis identified by the study included [1] the identification of key SRL processes with high inter-rater reliability [2] the identification of differences in key SRL processes between successful and unsuccessful students in strategic planning and self-monitoring [3] less than 5 min of student and observer time were sufficient to obtain useful information on the use of key SRL processes. The rationale and methods used in our pilot study can inform future research, and we recommend increasing the sample size and expand to a range of different clinical skills to investigate whether our findings may be generalized and also the potential of the findings to inform feedback.

## Conclusions

Our findings suggest that SRL microanalysis is a potentially useful approach to identify students’ use of key SRL processes during performance of clinical examination skills in physiotherapy. As this was a pilot study, further research with the same research design is recommended to ensure generalization as well as the reproducibility of our findings.

## Data Availability

All data generated or analysed during this study are included in this published article and its supplementary information are available from the corresponding author on reasonable request.

## Abbreviations

SRL: Self-regulated learning
RM: Raquel Medina
DA: David Álamo
MJC: Manuel João Costa
SPSS: Statistical Package for the Social Sciences
SE: self-efficacy
